# A155 ASEPTIC ABSCESS SYNDROME IN INFLAMMATORY BOWEL DISEASE: A CASE REPORT

**DOI:** 10.1093/jcag/gwab049.154

**Published:** 2022-02-21

**Authors:** L Tam, D Akhtar, B Salh

**Affiliations:** 1 The University of British Columbia Faculty of Medicine, Vancouver, BC, Canada; 2 Medicine, The University of British Columbia Faculty of Medicine, Vancouver, BC, Canada; 3 Vancouver General Hospital, Vancouver, BC, Canada

## Abstract

**Background:**

Aseptic abscess syndrome (AAS) is a rare extraintestinal manifestation of inflammatory bowel disease (IBD) and it is characterized by the formation of sterile collections of neutrophils in various organs. Misdiagnosis frequently leads to unnecessary surgical procedures and prolonged courses of antibiotics. There are only 61 reportable cases of AAS in the international literature and 2 cases in Canada. This case and the associated literature review highlight the diagnostic considerations, treatment options and disease course of this rare disease.

**Aims:**

To describe a case of AAS in IBD.

**Methods:**

Case report and review of the literature.

**Results:**

A 24-year-old male with a history of ulcerative pancolitis (UC) initially presented with a 3-week history of malaise, abdominal pain, and fevers with a computed tomography (CT) scan showing multiple splenic abscesses. Over the next year, he had multiple readmissions for recurrent sepsis from culture negative multi-focal abscesses. Serial CT scans revealed new abscesses in the liver, scrotum, and lungs with interval progression in size. Unsuccessful attempts to isolate a culprit organism which included serological markers and cultures from blood, bronchial washings, aspirate from abscesses, and surgical tissue. All samples demonstrated frank pus with suppurative granulomatous inflammation. The patient was trialed on various courses of antibiotics without improvement. Therefore, a decision was made to treat for AAS given his IBD history. Antibiotics were discontinued and prednisone was initiated with rapid improvement in symptoms and biochemistry. In the three years following, he was transitioned to a combination of infliximab and azathioprine. He had rapid resolution of his abscesses on serial CT scans following steroid and anti-TNF therapy. Currently, he is no longer on steroids and his CRP has normalized with no recurrence of his symptoms.

**Conclusions:**

AAS has often been associated with IBD, particularly Crohn’s disease and more rarely in UC. Case series have shown that AAS is not strongly associated with IBD activity as only 55% of cases have concomitant IBD flare and abscess formation. The syndrome continues to pose a diagnostic conundrum among specialists as there is no established diagnostic criteria and thus AAS remains a diagnosis of exclusion. This has led many patients to suffer from AAS for prolonged periods and receive unnecessary investigations and treatments. Steroids remain the gold standard for initial induction of remission. However, the ideal regimen for maintenance therapy is still evolving and case reports have described the effects of immunotherapy and biologics on AAS. AAS has a substantial impact on quality of life and is very responsive to therapy; this case report reminds the clinician that AAS should be considered in patients with IBD presenting with multifocal abscesses unresponsive to antimicrobials.

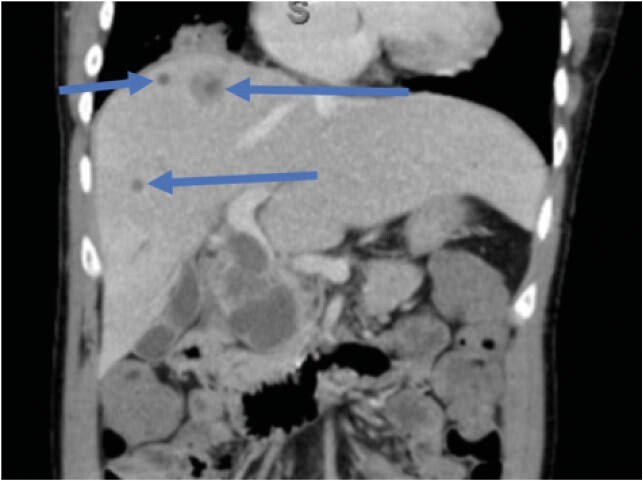

Serial CT scan (coronal view) of abdomen demonstrating multiple hepatic abscesses (blue arrows)

**Funding Agencies:**

None

